# Comparative evaluation of three anti-dsDNA antibody detection methods in systemic lupus erythematosus: insights from a large monocentric cohort

**DOI:** 10.3389/fimmu.2025.1529484

**Published:** 2025-04-10

**Authors:** Ruijing Lu, Rui Yu, Rong Huang, Chiyuan Xue, Ning Song, Jiuliang Zhao, Xiaofeng Zeng, Chaojun Hu

**Affiliations:** ^1^ Department of Rheumatology and Clinical Immunology, Peking Union Medical College Hospital, Chinese Academy of Medical Sciences, Peking Union Medical College, National Clinical Research Center for Dermatologic and Immunologic Diseases (NCRC-DID), Ministry of Science & Technology, Key Laboratory of Rheumatology and Clinical Immunology, Ministry of Education, Beijing, China; ^2^ Department of Laboratory Medicine, Shenzhen Baoan Women’s and Children’s Hospital, Shenzhen, China; ^3^ Eight-year Medical Doctor Program, Chinese Academy of Medical Sciences & Peking Union Medical College, Beijing, China; ^4^ Department of Clinical Laboratory, The People’s Hospital of Yuhuan, Taizhou, Zhejiang, China

**Keywords:** anti-double-stranded DNA, systemic lupus erythematosus, indirect immunofluorescence assay, digital liquid chip method, chemiluminescence immunoassay

## Abstract

**Background:**

Anti-double-stranded DNA (anti-dsDNA) antibodies at abnormal titer are of considerable diagnostic value for systemic lupus erythematosus (SLE). Current assays detecting anti-dsDNA antibodies show divergent properties, emphasizing the importance of selecting suitable assays. This study aims to investigate the diagnostic performance of indirect immunofluorescence (IIF), digital liquid chip method (DLCM), chemiluminescence immunoassay (CLIA), and their combinations for detecting anti-dsDNA antibodies in SLE.

**Methods:**

We conducted a retrospective, single-center study from 2022 to 2023 which included 3429 samples: 1773 from patients with SLE and 1656 from controls with rheumatoid arthritis (RA) and Sjögren’s syndrome (SS). Sensitivity, specificity, accuracy, positive predictive value (PPV), and negative predictive value (NPV) for anti-dsDNA detection by IIF, DLCM, and CLIA were calculated. Cohen’s kappa coefficient was used to evaluate inter-method agreement. The correlations between anti-dsDNA concentration and SLEDAI-2k scores/renal involvement were assessed.

**Results:**

Among individual assays, IIF demonstrated the highest specificity (98.31%) and PPV (96.10%) but lower sensitivity (38.92%) compared to CLIA (41.57%) and DLCM (43.65%) (p < 0.05). Combining two assays significantly improved sensitivity while maintaining specificity>95%. The combination of IIF and DLCM achieved a sensitivity of 52.2% and an AUC of 0.76. Substantial agreement was observed between DLCM and CLIA (κ = 0.78), whereas agreement between IIF and the other assays was moderate (κ = 0.65–0.66). In a longitudinal analysis of 88 SLE patients, CLIA and DLCM detected antibody fluctuations more reliably than IIF. Anti-dsDNA levels by DLCM or CLIA positively correlated with SLEDAI-2K scores (R=0.42 and 0.29, p<0.05). Both IIF and CLIA methods showed significant differences between the SLE patients with and without renal involvement (p < 0.05). The combination of two assays provided higher sensitivity than single assays (p<0.001) in renal involvement subgroups.

**Conclusion:**

Our findings demonstrate that DLCM performs comparably to CLIA, supporting its clinical potential. Moreover, combining assays significantly enhances diagnostic sensitivity, particularly in subgroups with renal involvement.

## 1 Introduction

Systemic lupus erythematosus (SLE) is a complex autoimmune disease characterized by the production of autoantibodies against nuclear components, leading to multi-organ involvement and diverse clinical manifestations (1). Among these autoantibodies, anti-double-stranded DNA (anti-dsDNA) antibodies are important for SLE and are integral to its diagnosis and classification (2). Although anti-dsDNA antibodies are not standalone diagnostic biomarkers for SLE, they are included as a key immunological criterion in the 2019 European League Against Rheumatism (EULAR)/American College of Rheumatology (ACR) classification criteria for SLE (3, 4). The presence of anti-dsDNA antibodies often precedes clinical symptoms and is associated with disease severity, particularly renal involvement such as glomerulonephritis (5–7).

However, the conception of anti-dsDNA antibodies as a homogeneous and highly specific biomarker for SLE is an oversimplification that overlooks the complexity of their antigenic diversity and the heterogeneity of patient responses (8–10). Anti-dsDNA antibodies constitute a diverse group targeting multiple DNA structures, including B-DNA, Z-DNA, single-stranded DNA (ssDNA), RNA-DNA hybrids, and various DNA-protein complexes (11–13). This antigenic diversity contributes to the variability in their detection and the interpretation of their clinical significance (14).

The detection of anti-dsDNA antibodies is further complicated by the variability among laboratory assays (15). Different detection methods—including indirect immunofluorescence (IIF), chemiluminescence immunoassay (CLIA), and digital liquid chip method (DLCM)—employ various antigenic sources and detection principles, leading to discrepancies in sensitivity and specificity. IIF using *Crithidia luciliae* offers high specificity but limited sensitivity (16), while CLIA provides quantitative results with higher sensitivity but may exhibit cross-reactivity (17). DLCM, a novel multiplex assay technology, integrates microfluidic technology with digitally encoded magnetic beads, enabling simultaneous quantification of multiple analytes from a single microliter-volume sample. While the application of DLCM in anti-dsDNA antibody detection holds significant promise, current research is limited and further validation is required to fully establish its clinical utility (18).

Given these complexities, there is a pressing need to evaluate the concordance between different anti-dsDNA antibody detection methods (19). Since double-screening strategy has been recommended by a proposal from a Spanish expert panel (20), it is necessary to assess their combined diagnostic utility. Previous studies have highlighted the importance of assay selection and the potential benefits of employing multiple assays to capture the full spectrum of anti-dsDNA antibodies (21, 22). By exploring the strengths and limitations of each method, clinicians can better interpret test results and tailor diagnostic strategies to individual patients.

The primary objective of this study was to compare the diagnostic performance of IIF, CLIA, and DLCM assays in detecting anti-dsDNA antibodies among patients with SLE. We aimed to assess the concordance between these methods and evaluate whether combining assays enhances diagnostic accuracy. Additionally, we will investigate the relationship between different testing strategies and disease activity as well as renal involvement. By identifying the most reliable approach for anti-dsDNA antibody detection, this study seeks to improve diagnostic precision and contribute to better clinical outcomes for patients with SLE.

## 2 Methods

### 2.1 Study design and patient population

This single-center, retrospective study screened serum samples from consecutively submitted laboratory samples at Peking Union Medical College Hospital between July 2022 and July 2023. Patients were eligible if they fulfilled the 1997 ACR criteria (6), the 2012 Systemic Lupus International Collaborating Clinics (SLICC) classification criteria (23) or 2019 EULAR/ACR criteria (24). Additionally, patients diagnosed with Sjögren’s syndrome (SS) according to the 2016 ACR/EULAR classification criteria (25), or rheumatoid arthritis (RA) according to the 2010 ACR/EULAR classification criteria (26) were included as disease control groups. In brief, 3081 patients were enrolled, comprising 1509 SLE patients, 662 SS patients, and 910 RA patients. A total of 3429 serum samples were collected, including 1773 from SLE patients, 937 from RA patients, and 719 from SS patients.

Clinical information at baseline was extracted from patient medical records, including age, sex, antinuclear antibodies (ANA) titer and fluorescence pattern, and other laboratory abnormalities. Moreover, 351 SLE patients had detailed clinical assessments which had been reported previously (27), including organ manifestations and SLEDAI-2000 score. This study was approved by the Medical Ethics Committee of PUMCH (Beijing, China) and written informed consent was obtained from all participants before inclusion in the study.

### 2.2 Sample collection and anti-dsDNA antibody assays

Serum samples were collected from all patients and stored at –80°C and were thawed only once before testing to preserve antibody integrity. The anti-dsDNA IgG antibodies were measured using three different immunoassays performed according to the manufacturer’s instructions.

#### 2.2.1 Indirect immunofluorescence assay

The IIF assay was conducted using the *Crithidia luciliae* indirect immunofluorescence test kit (EUROIMMUN AG, Lübeck, Germany). Slides coated with *C. luciliae* were incubated with diluted sera. In case of a positive sample, specific IgG, IgA, and IgM antibodies bind to the flagellate antigen. During the second incubation step, the fluorescein-labeled anti-human IgG antibody interacts with the antibodies bound to the biological matrix. Slides were then washed, mounted with a coverslip, and examined under a fluorescence microscope) A positive result was indicated by bright fluorescence of the kinetoplast.

#### 2.2.2 Chemiluminescent immunoassay

The CLIA was performed using the iFlash 3000 chemiluminescence immunoanalyzer (YHLO Biotech Co., Ltd., Shenzhen, China). Prediluted serum samples were mixed with biotin-labeled dsDNA antigen and streptavidin-coated magnetic microparticles. The dsDNA antibodies in the samples bound to the antigen-coated microparticles. After incubation and washing steps to remove unbound substances, an acridinium ester–labeled anti-human IgG secondary antibody was added. The relative luminescence intensity (RLU) was measured, and results equal to or greater than 36 IU/mL were considered positive, as per the manufacturer’s cutoff values.

#### 2.2.3 Digital liquid chip method

The DLCM assay was carried out using the MCLIA-800 system (Livzon Diagnostics Inc, Guangdong, China). This multiplex assay employed lyophilized barcoded magnetic beads (BMBs) conjugated with dsDNA antigens (Applied BioCode Inc., Santa Fe Springs, CA, USA). Upon reconstitution, the antigen-coated BMBs were incubated with diluted serum samples for 30 minutes at room temperature. After washing, phycoerythrin-conjugated anti-human IgG antibodies were added and incubated for an additional 30 minutes. The beads were then washed, and the median fluorescence intensity (MFI) was measured using the BioCode MDx-3000 system. An antibody index of ≥1.0 was considered positive according to the manufacturer’s guidelines.

### 2.3 Statistical analysis

Based on the manufacturer’s recommended cut-off values, sensitivity, specificity, positive predictive value (PPV), and negative predictive value (NPV) were calculated for each assay and their respective combinations. McNemar’s test was utilized to compare the paired proportions between the diagnostic methods. Receiver operating characteristic (ROC) curves were generated to evaluate the diagnostic accuracy of each assay, and the area under the curve (AUC) was calculated. The inter-method agreement was assessed using Cohen’s kappa coefficient (κ), which accounts for agreement occurring by chance. Pearson correlation analysis was used to explore the correlation between anti-dsDNA concentration and SLEDAI-2k scores. Comparisons between groups were made using the Mann-Whitney U test or the Wilcoxon signed-rank test, as appropriate. A two-tailed p-value of <0.05 was considered statistically significant. Statistical significance was defined as a two-tailed p-value of <0.05. All statistical analyses were performed using SPSS software (version 26.0.0.0; IBM Corp., Armonk, NY, USA) and Python (version 3.11.8) with the sklearn.metrics module.

## 3 Results

### 3.1 Patients characteristics

A total of 3081 patients were included in the analysis, comprising 1509 patients with SLE and 1572 other autoimmune disease controls (662 with SS and 910 with RA). The baseline demographic and clinical characteristics of the study population are summarized in **Table 1**. The mean age of patients with SLE was 33.98 ± 14.44 years, which was significantly younger than that of the control group (48.19 ± 14.28 years; p < 0.05). A female predominance was observed across all groups, consistent with the epidemiology of autoimmune diseases. Among SLE patients, 96.62% tested positive for ANA, predominantly exhibiting speckled (70.6%) or homogeneous (64.6%) patterns. The ANA titers across SLE patients are presented in **Supplementary Table 1**. Furthermore, the prevalence of anti-RNP, anti-Sm and anti-rRNP antibodies was significantly elevated in SLE patients (p<0.001).

**Table 1:** Baseline demographic characteristics of study participants.

Groups	SLE	RA	SS	Controls
Number	1509	910	662	1572
Age, Mean ± SD, y	33.98 ± 14.44^†^	49.37 ± 13.49	46.57 ± 15.17	48.19 ± 14.28^†^
Female, n (%)	1362 (90.26)	802 (88.13)	634 (95.77)	1436 (91.35)
Samples	1773	937	719	1656
ANA patterns, n (%)				
Speckled	878 (49.52)	163 (17.40)	522 (72.60)	685 (41.36)
Homogeneous	489 (27.58)	393 (41.94)	51 (7.09)	444 (26.81)
Nucleolar	17 (0.96)	8 (0.85)	2 (0.28)	10 (0.60)
Cytoplasmic	27 (1.52)	26 (2.77)	14 (1.95)	40 (2.42)
Homogeneous-Speckled	270 (15.23)	76 (8.11)	50 (6.95)	126 (7.61)
Speckled-Nucleolar	15 (0.85)	10 (1.07)	22 (3.06)	32 (1.93)
Homogeneous-Nucleolar	14 (0.79)	12 (1.28)	5 (0.70)	17 (1.03)
Other rare patterns*	3 (0.17)	11 (1.17)	6 (0.83)	17 (1.03)
Negative	60 (3.38)	238 (25.40)	47 (6.54)	285 (17.21)
Laboratory abnormalities, n (%)				
Anti-RNP (+)	550 (31.02)	23 (2.45)	44 (6.12)	67 (4.05)
Anti-Sm (+)	393 (22.17)	5 (0.53)	16 (2.23)	21 (1.27)
Anti-SSA (+)	966 (54.48)	107 (11.42)	586 (81.50)	693 (41.85)
Anti-SSB (+)	276 (15.57)	31 (3.31)	243 (33.80)	274 (16.55)
Anti-rRNP (+)	518 (29.22)	18 (1.92)	27 (3.76)	45 (2.72)

† indicate statistically significant differences between the age of SLE patients and controls (p<0.05).

* Other rare patterns include wave protein, spindle shape, nuclear membrane, ring (rod) shape, and myosin (myofibrillar).

SLE, systemic lupus erythematosus; RA, rheumatoid arthritis; SS, Sjögren’s syndrome; RNP, ribonucleoprotein; Sm, Smith; SSA, Sjögren’s Syndrome Antigen A; SSB, Sjögren’s Syndrome Antigen B.

Among the 351 SLE patients with detailed clinical assessments, 398 samples were analyzed. Organ involvement and disease activity were presented in **Table 2**. The mean age of the 351 patients was 34.46 ± 11.86 years, which was comparable to the overall SLE cohort. Cutaneous (281, 70.60%) and hematologic (257, 64.57%) manifestations were most frequent. Renal involvement was observed in 143 samples (35.93%). Disease activity, as assessed by SLEDAI-2k scores, was predominantly mild (score ≤6) in 302 samples (75.88%). Moderate activity (score 7–12) and severe activity (score >12) were observed in 65 (16.33%) and 31 samples (7.79%), respectively.

**Table 2:** Organ involvement and SLEDAI-2k scores within 351 SLE patients.

	SLE patients (n=351, samples=398)
Age, Mean ± SD, y	34.46 ± 11.86
Female, n (%)	332 (94.59)
Organ involvement, n (%)*	
Renal Involvement	143 (35.93)
Cutaneous Involvement	281 (70.60)
Musculoskeletal Involvement	173 (43.37)
Cardiovascular Involvement	24 (6.03)
Pulmonary Involvement	11 (2.76)
Neurological Involvement	50 (12.56)
Hematologic Involvement	257 (64.57)
Gastrointestinal Involvement	0 (0.00)
SLEDAI-2k scores, n (%)	
Mild (≤6)	302 (75.88)
Moderate (7-12)	65 (16.33)
Severe (>12)	31 (7.79)

* The classification of organ involvement was based on the SLEDAI-2k scoring criteria. Renal involvement included heme-granular or RBC urinary casts, hematuria, proteinuria, and pyuria. Cutaneous involvement included inflammatory-type rash, alopecia, oral or nasal mucosal ulcers, and vasculitis. Musculoskeletal involvement included arthritis and myositis. Cardiovascular involvement included pericarditis, myocarditis, and endocarditis. Pulmonary involvement included pleuritis, pneumonitis, interstitial lung disease, and pulmonary hypertension. Neurological involvement included seizure, psychosis, organic brain syndrome, new onset sensory or motor neuropathy involving cranial nerves, lupus headache, and new onset stroke. Hematologic Involvement included anemia, leukopenia, thrombocytopenia, and lymphadenopathy. Gastrointestinal Involvement included nausea, vomiting, abdominal pain, and mesenteric vasculitis or pancreatitis.

### 3.2 Diagnostic performance of individual assays and their combinations

**Figure 1:**
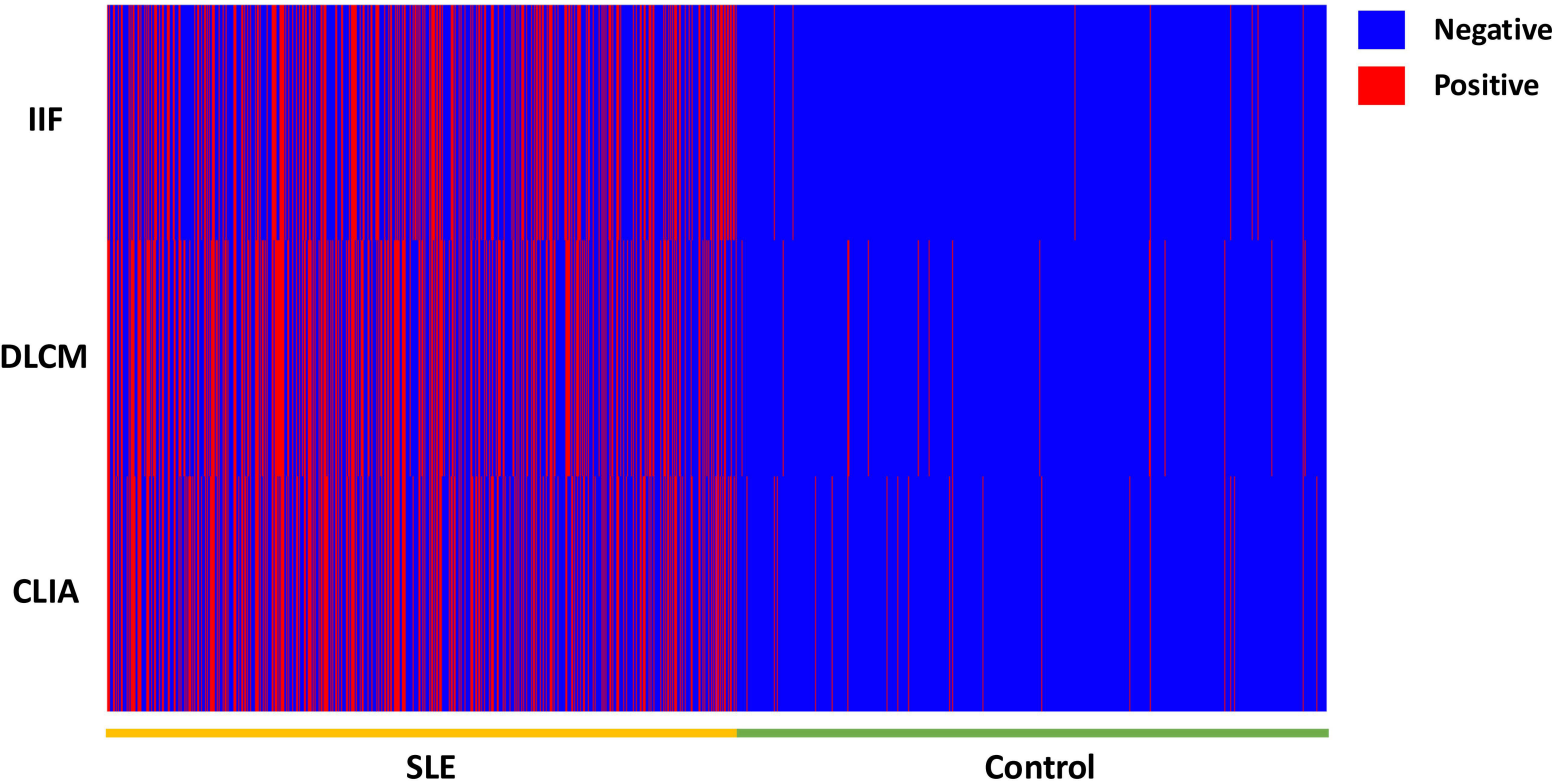
The heat map of the anti-dsDNA antibody detection result by IIF, CLIA, and DCLM in 3429 samples.

The diagnostic performance of individual and combined anti-dsDNA antibody detection methods is presented in **Table 3** and **Supplementary Table 2**. All three methods performed high specificity, as depicted in **Figure 1**. The IIF assay demonstrated the highest specificity (98.31%) and PPV (96.10%); however, its sensitivity (38.92%) was significantly lower compared to DLCM (43.65%) and CLIA (41.57%) (p < 0.05). DLCM exhibited the highest sensitivity and accuracy (69.29%) with a specificity of 96.7%. NPVs for all three methods were relatively similar, ranging from 60.05% to 61.59%.

**Table 3:** Clinical performance of IIF, CLIA, DLCM and their combinations in anti-dsDNA antibodies detection.

Assay	SLE	Control	Sensitivity	Specificity	Accuracy	PPV	NPV	AUC	95% CI
IIF	690 (38.92%)	28 (1.69%)	38.92%^b,c^	98.31%^b,c^	67.60%^b,c^	96.10%	60.05%	0.69	[0.665,0.708]
DLCM	774 (43.65%)	54 (3.26%)	43.65%^a,c^	96.74%^a^	69.29%^a,c^	93.48%	61.59%	0.71	[0.684,0.728]
CLIA	737 (41.57%)	57 (3.44%)	41.57%^a,b^	96.56%^a^	68.12%^a,b^	92.82%	60.68%	0.69	[0.671,0.715]
IIF/DLCM*	926 (52.23%)	68 (4.11%)	52.23%^a,b,c^	95.89%^a,b^	73.32%^a,b,c^	93.16%	65.22%	0.76	[0.736,0.781]
IIF/CLIA*	904 (50.99%)	93 (5.62%)	50.99%^a,b,c^	94.38%^a,c^	71.95%^a,b,c^	90.67%	64.27%	0.75	[0.728,0.772]
DLCM/CLIA*	904 (50.99%)	72 (4.35%)	50.99%^a,b,c^	95.65%^a,b^	72.56%^a,b,c^	92.62%	64.57%	0.74	[0.713,0.757]
IIF/DLCM/CLIA*	1000 (56.40%)	104 (6.28%)	56.40%^a,b,c^	93.72%^a,b,c^	74.42%^a,b,c^	90.58%	66.75%	0.77	[0.751,0.794]

* Combinations refer to patients who tested positive by any of the included methods (e.g., IIF/DLCM indicates patients positive by either DLCM or IIF).

The superscript letters (a, b, c) indicate statistically significant differences compared to IIF, DLCM, and CLIA methods, respectively (p < 0.05). Specific comparisons and P-values are detailed in the **Supplementary Table 2**.

SLE, systemic lupus erythematosus; IIF, indirect immunofluorescence; CLIA, chemiluminescence immunoassay; DLCM, digital liquid chip method; PPV, positive predictive value; NPV, negative predictive value; AUC, area under curve; CI, confidence interval.

In contrast, combining two assays led to a statistically significant improvement in sensitivity compared to any single assay alone. As shown in **Table 3**, the combination of IIF and DLCM resulted in a sensitivity of 52.23% (p < 0.001 vs. IIF) and a specificity of 95.89%. The IIF/CLIA combination followed closely with a sensitivity of 50.99% and an accuracy of 71.95%. Notably, combining all three assays increased sensitivity to 56.40%, while specificity slightly decreased to 93.72%.

ROC curve analysis confirmed these findings. The AUC for DLCM was 0.71 (95% CI [0.68, 0.73]), indicating moderate diagnostic accuracy, while AUCs for IIF and CLIA were both 0.69. Combining IIF and DLCM increased the AUC to 0.76, and the combination of all three assays yielded the highest AUC of 0.77 (95% CI [0.75, 0.79]), underscoring the diagnostic advantage of assay combinations.

### 3.3 Distribution of anti-dsDNA antibody positivity across detection methods

**Figure 2:**
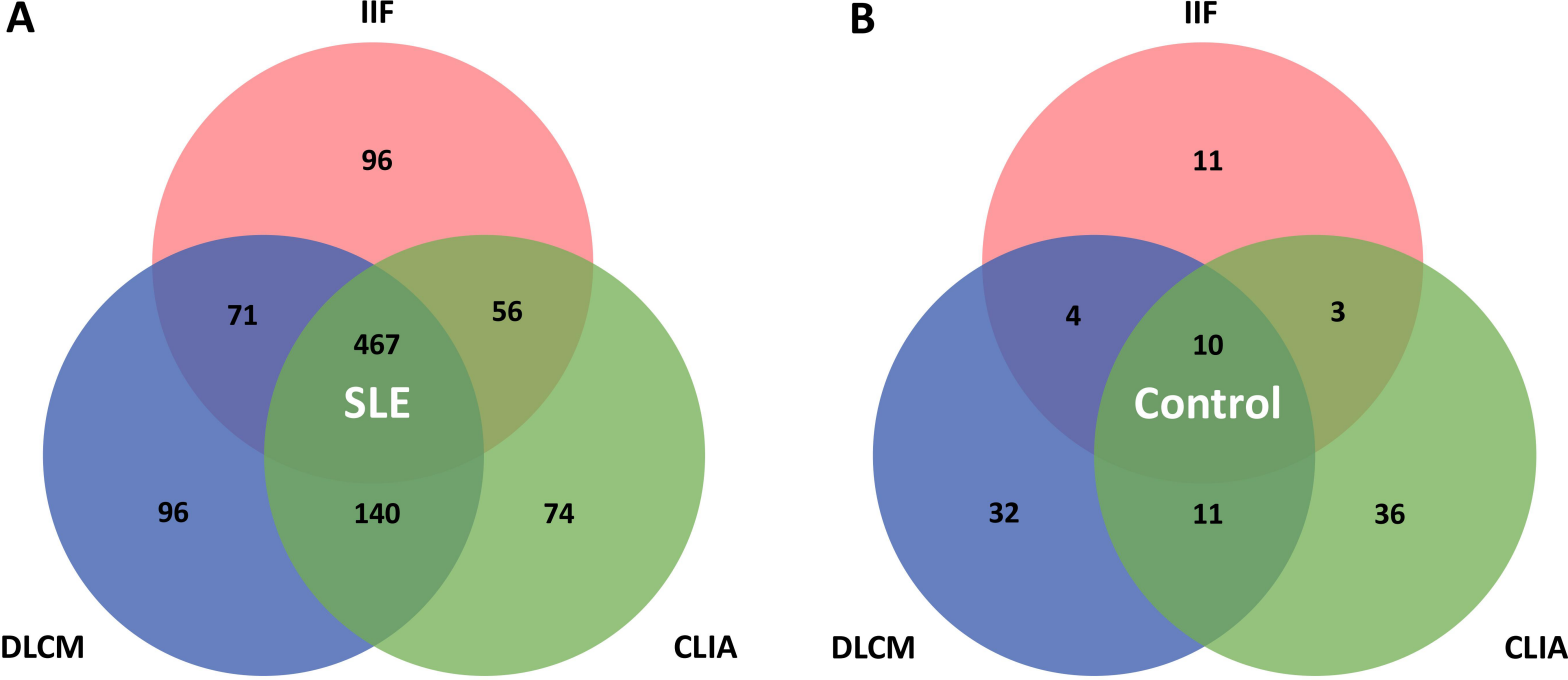
Venn diagrams illustrate the overlap between IIF, CLIA, and DLCM detection assays. **(A, B)** show the distribution of positivity for the three assays in patients with SLE and controls with other autoimmune diseases, respectively.

Various patterns of assay positivity were observed and the distribution of positivity across the three detection methods is detailed in **Table 4** and illustrated in **Figure 2**. Among the 1773 SLE samples, 467 (26.3%) tested positive for anti-dsDNA antibodies by all three methods, whereas only 10 control subjects (0.6%) exhibited triple positivity. Additionally, 96 patients (5.4%) were positive exclusively by IIF, another 96 (5.4%) solely by DLCM, and 74 (4.2%) only by CLIA. These patterns imply that each assay would capture distinct subsets of anti-dsDNA antibodies. Combining multiple detection methods enhances the overall sensitivity and diagnostic yield for identifying patients with systemic lupus erythematosus. The Venn diagrams in **Figure 2** depict the overlap of positive results among the three assays for both SLE patients and control subjects.

**Table 4:** Distribution of anti-dsDNA antibody positivity across IIF, CLIA, and DLCM methods.

Detection Pattern (IIF/DLCM/CLIA)	SLE (n=1773)	Controls (n=1656)	Total (n=3429)
**-/-/-**	773 (43.60%)	1552 (93.72%)	2325 (67.80%)
**-/-/+**	74 (4.17%)	36 (2.17%)	110 (3.21%)
**-/+/-**	96 (5.41%)	32 (1.93%)	128 (3.73%)
**+/-/-**	96 (5.41%)	11 (0.66%)	107 (3.12%)
**-/+/+**	140 (7.90%)	8 (0.48%)	148 (4.32%)
**+/-/+**	56 (3.16%)	3 (0.18%)	59 (1.72%)
**+/+/-**	71 4.00%)	4 (0.24%)	75 (2.19%)
**+/+/+**	467 (26.34%)	10 (0.60%)	477 (13.91%)

“+” indicates a positive result; “−” indicates a negative result.

IIF, indirect immunofluorescence; CLIA, chemiluminescence immunoassay; DLCM, digital liquid chip method.

### 3.4 Concordance among the three detection assays

The concordance between the detection assays was assessed using Cohen’s kappa coefficients. There was substantial agreement between DLCM and CLIA (κ = 0.78), indicating a high level of consistency in their detection of anti-dsDNA antibodies. In contrast, the agreement between IIF and DLCM was moderate (κ = 0.65), as was the agreement between IIF and CLIA (κ = 0.66). These results suggest that while DLCM and CLIA have similar detection capabilities, IIF may identify more different antibody subsets.

### 3.5 Hierarchical distribution of anti-dsDNA antibody levels

**Figure 3:**
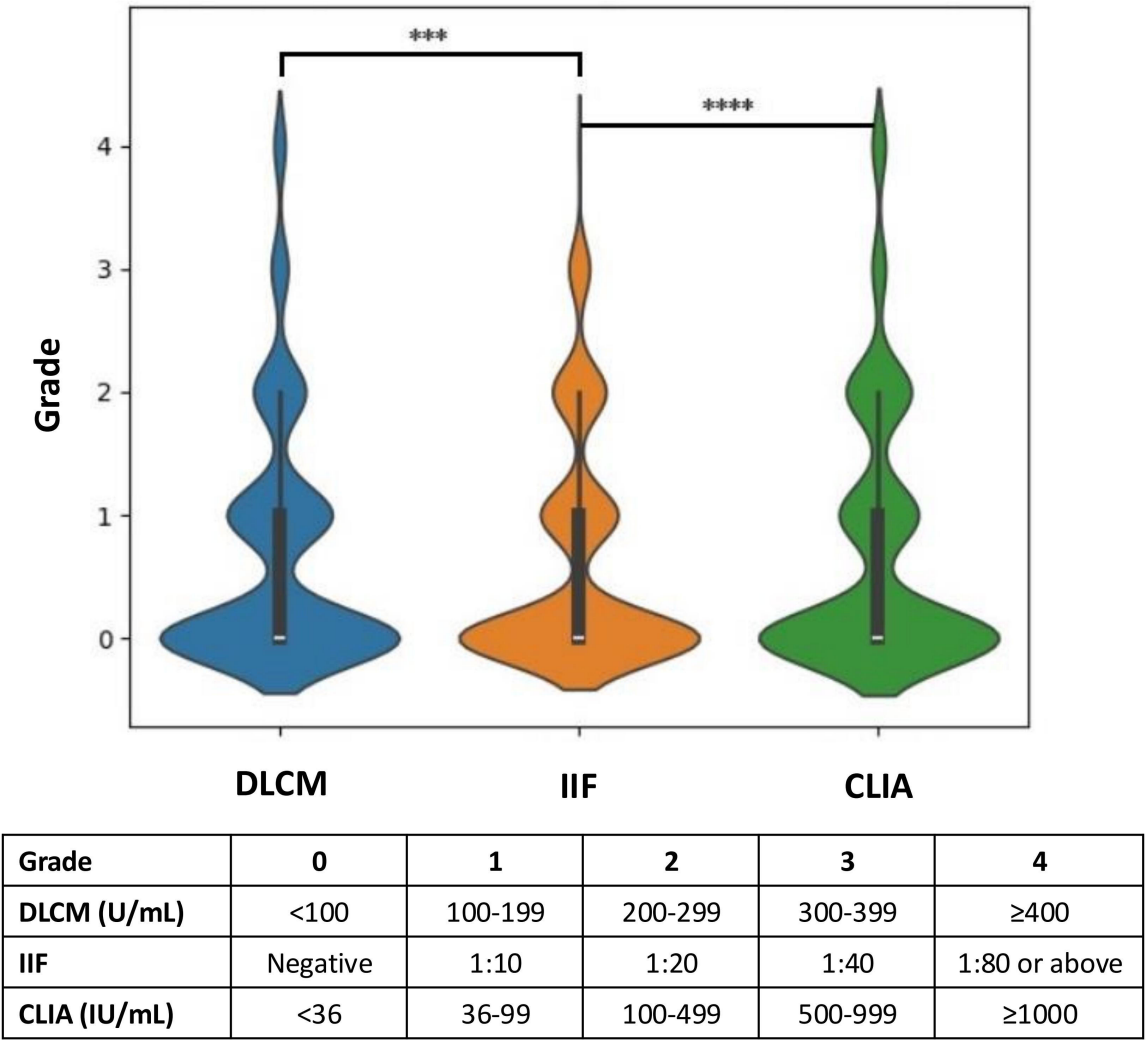
Hierarchical distribution of anti-dsDNA antibody levels detected by CLIA, DLCM, and IIF. The results of the three assays are presented in hierarchical form based on specific grading rules for each method. Higher grades correspond to higher concentrations or titers of anti-dsDNA antibodies. Statistical significance is indicated as ****p < 0.0001; ***p < 0.001.

**Figure 3** illustrates the hierarchical distribution of anti-dsDNA antibody levels detected by the three assays in relation to the clinical diagnoses. The assay results were stratified into grades from 0 to 4 based on specific concentration ranges to facilitate comparison across methods. Analysis revealed that higher-grade antibody levels (grades 3 and 4) were more frequently observed in CLIA and DLCM compared to IIF (p < 0.001). Specifically, the proportion of patients with high antibody titers was significantly greater in CLIA and DLCM assays than in the IIF assay, indicating that CLIA and DLCM are more sensitive in detecting elevated levels of anti-dsDNA antibodies.

### 3.6 Longitudinal changes in anti-dsDNA antibody levels detected by different methods

To evaluate the consistency of the detection methods in reflecting changes in anti-dsDNA antibody levels over time, we analyzed data from 88 SLE patients who underwent two serial anti-dsDNA antibody tests using all three assays during the study period. For each patient, the change in antibody titer grade between the two tests was calculated for each method by subtracting the initial grade from the subsequent grade, based on predefined grading criteria specific to each assay (as previously described).

**Figure 4:**
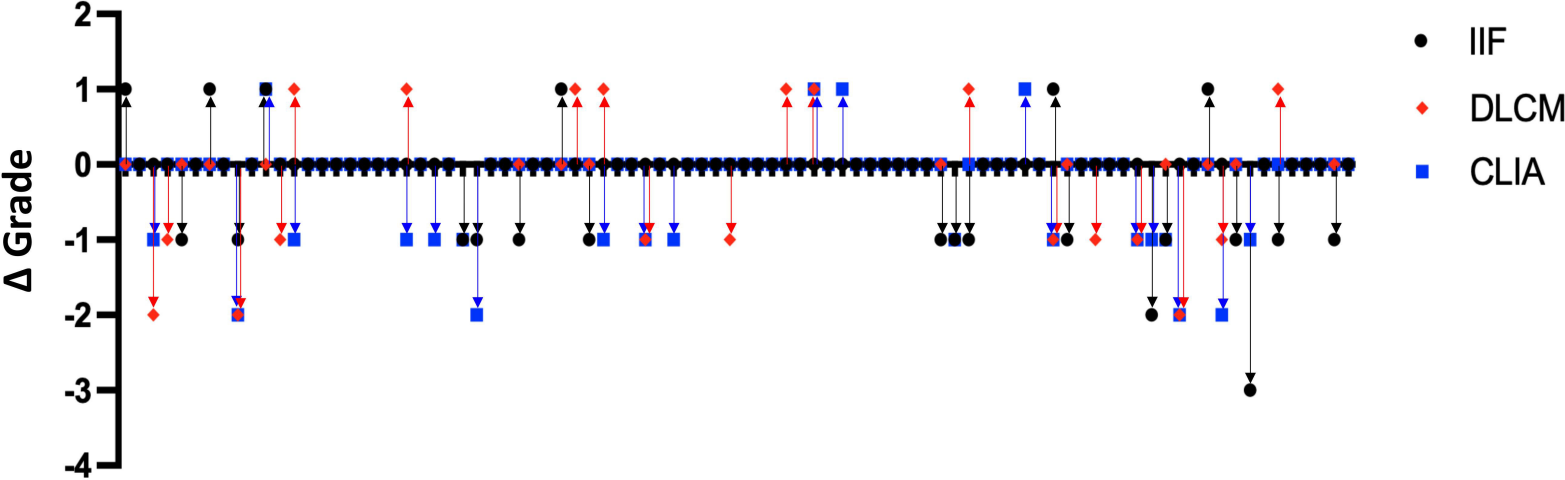
Consistency of anti-dsDNA antibody titer changes detected by IIF, CLIA, and DLCM methods. Dot plots showing the changes in anti-dsDNA antibody titer grades between consecutive tests for 88 SLE patients, as measured by IIF (black circles), CLIA (blue squares), and DLCM (red diamonds). Each point represents the change in titer grade for a single patient, calculated by subtracting the initial grade from the subsequent grade for each method.

**Figure 4** illustrates the distribution of titer grade changes for each assay. The majority of patients exhibiting concordant grade increases or decreases between consecutive tests. However, CLIA and DLCM more consistently detected changes in antibody levels. In contrast, the IIF method displayed greater variability. CLIA and DLCM might track antibody changes more reliably in SLE monitoring

### 3.7 Correlation between assay-specific anti-dsDNA concentrations and disease activity

**Figure 5:**
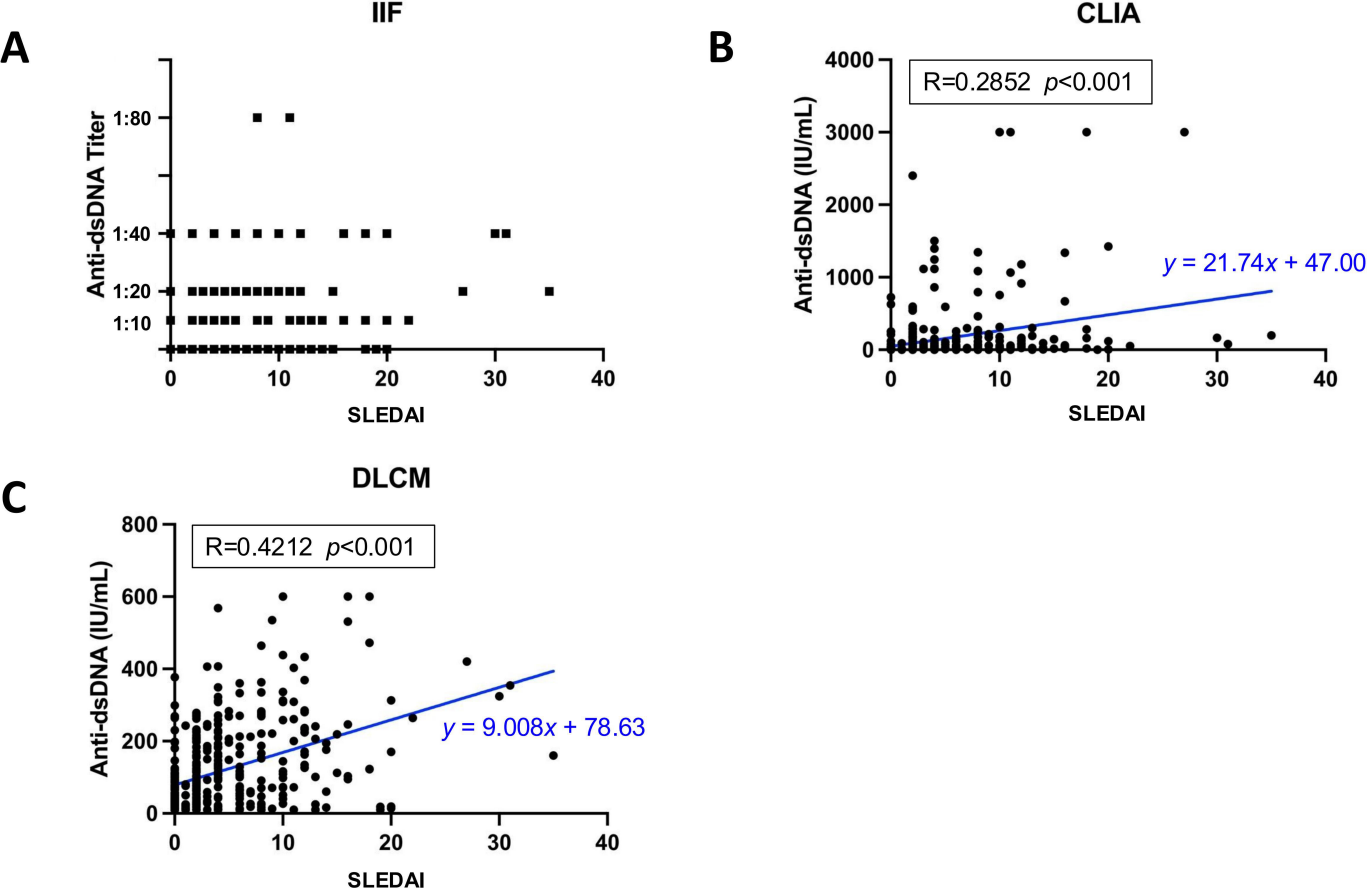
Correlation between assay-specific anti-dsDNA concentrations and disease activity. **(A)** Distribution of anti-dsDNA titers detected by IIF in relation to SLEDAI scores. **(B, C)** show the Pearson’s correlations for anti-dsDNA concentrations detected by CLIA or DLCM and SLEDAI scores, respectively. The correlation coefficient (R) and statistical significance (p) values are provided, along with the regression line equation.

Among 398 SLE samples with detailed clinical information, the association of anti-dsDNA antibody levels detected by each assay with SLE disease activity was evaluated (**Figure 5**). Disease activity was quantified using the SLEDAI-2k score. The median anti-dsDNA titers assessed by IIF was negative (IQR [-,1:10]) in mild patients (SLEDAI-2k ≤ 6), and 1:10 (IQR [-,1:20]) in medium and severe patients. Pearson’s correlation analysis revealed a moderate positive correlation between anti-dsDNA concentrations measured by DLCM and SLEDAI scores (R = 0.4212, p < 0.001). Similarly, CLIA-derived anti-dsDNA levels showed a positive correlation with SLEDAI scores, though the association was weaker than that observed with DLCM (R = 0.2852, p = 0.015).

### 3.8 Association of assay-specific anti-dsDNA concentrations with renal involvement

**Figure 6:**
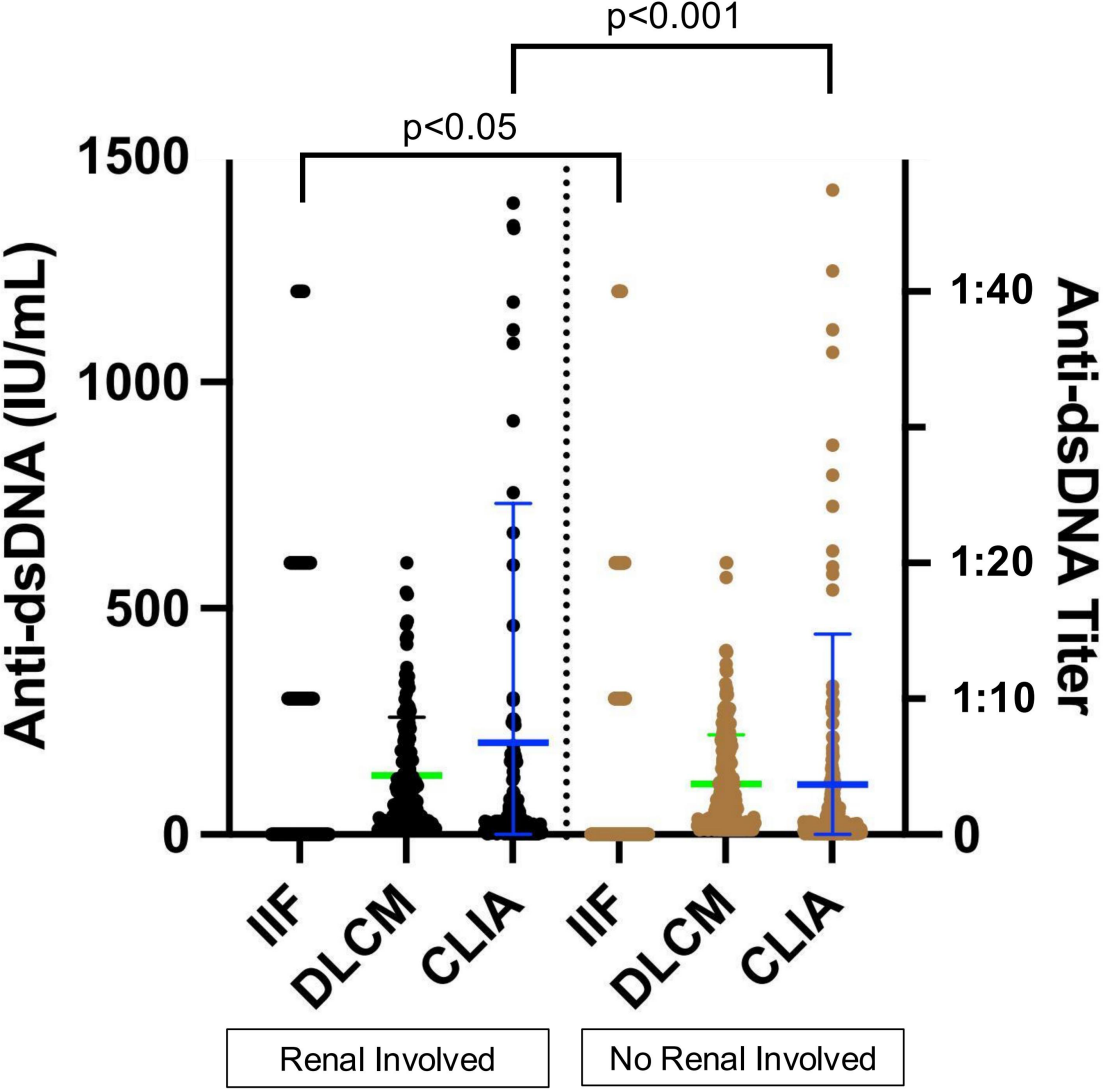
Association of assay-specific anti-dsDNA concentrations with renal involvement. Black and brown dots represent anti-dsDNA levels in serum samples from patients with and without renal involvement, respectively. The left y-axis corresponds to CLIA and DLCM measurements, while the right y-axis corresponds to IIF measurements. Green and blue lines indicate the mean ± SD for DLCM and CLIA, respectively.

Previous studies have reported an association between anti-dsDNA levels and renal involvement. Among the 398 SLE samples with detailed clinical information, 143 were from patients with renal involvement and 255 from those without (**Table 5**). Anti-dsDNA levels were compared between the two subgroups (**Figure 6**). Both IIF and CLIA methods showed significant differences between the subgroups (p < 0.05 and p < 0.001, respectively). However, quantitative DLCM measurements revealed no statistically significant difference in anti-dsDNA concentrations between the renal and non-renal groups (p = 0.132).

**Table 5:** Clinical performance of IIF, CLIA, DLCM and their combinations in anti-dsDNA antibodies detection in renal involvement subgroups.

Assay	SLE with Renal Involvement	SLE without Renal Involvement	Sensitivity	Specificity	Accuracy	PPV	NPV	AUC	95% CI
IIF	70 (48.95%)	79 (30.98%)	48.95%	69.02% ^b,c^	61.81%	46.98%	70.68%	0.59	[0.531,0.648]
DLCM	66 (46.15%)	108 (42.35%)	46.15%	57.65% ^a^	53.52%	37.93%	65.63%	0.52	[0.460,0.578]
CLIA	64 (44.76%)	96 (37.65%)	44.76%	62.35% ^a^	56.03%	40.00%	66.81%	0.54	[0.476,0.595]
IIF/DLCM*	81 (56.64%)	118 (46.27%)	56.64% ^a,b,c^	53.73% ^a,b,c^	54.77%	40.70%	68.84%	0.55	[0.493,0.611]
IIF/CLIA*	80 (55.94%)	115 (45.10%)	55.94% ^a,b,c^	54.90% ^a,c^	55.28%	41.03%	68.97%	0.55	[0.495,0.613]
DLCM/CLIA*	73 (51.05%)	126 (49.41%)	51.05% ^a,b,c^	50.59% ^a,b,c^	50.75%	36.68%	64.82%	0.51	[0.449,0.567]
IIF/DLCM/CLIA*	83 (58.04%)	131 (51.37%)	58.04% ^a,b,c^	48.63% ^a,b,c^	52.01%	38.79%	67.39%	0.53	[0.474,0.592]

* Combinations refer to patients who tested positive by any of the included methods (e.g., IIF/DLCM indicates patients positive by either DLCM or IIF).

The superscript letters (a, b, c) indicate statistically significant differences compared to IIF, DLCM, and CLIA methods, respectively (p < 0.05). Specific comparisons and P-values are detailed in the **Supplementary Table 3**.

SLE, systemic lupus erythematosus; IIF, indirect immunofluorescence; CLIA, chemiluminescence immunoassay; DLCM, digital liquid chip method; PPV, positive predictive value; NPV, negative predictive value; AUC, area under curve; CI, confidence interval.

The sensitivity of the three methods in the renal involvement group did not show significant differences, with IIF demonstrating the highest specificity (69.02%) (**Table 4**, **Supplementary Table 3**). Compared to single methods, dual combinations significantly improved sensitivity (e.g., IIF/DLCM 56.64% vs IIF 48.95%, p = 0.001). However, the specificity of the combinations decreased (e.g., IIF/DLCM 53.73% vs IIF 69.02%, p < 0.001), highlighting a trade-off between early detection and false positives.

## 4 Discussion

SLE is a complex autoimmune disorder with an insidious onset and variable clinical manifestations, often leading to diagnostic delays and misdiagnoses. Anti-dsDNA antibodies are key biomarkers for diagnosing SLE and monitoring disease activity, yet methodological variability across detection assays complicates their clinical interpretation (28). This study, the largest to date in a Chinese cohort (n=3081), systematically evaluates three anti-dsDNA detection methods—IIF, CLIA, and DLCM—and their combinations, offering critical insights for optimizing diagnostic workflows. Our findings confirm that while each method performs well individually, combining assays significantly improves diagnostic sensitivity without substantially compromising specificity. In addition, we found DLCM exhibited better correlation with SLEDAI-2k, while IIF and CLIA had the potential for renal-specific prognosis.

Our study demonstrates that IIF exhibits the highest specificity (98.31%), likely due to its use of native dsDNA as the target antigen (29). This native form of the antigen more accurately resembles the physiological target *in vivo* immune response compared to synthetic or recombinant antigens (30). However, its sensitivity (38.92%) was limited by subjective fluorescence interpretation and epitope disparities between kinetoplast DNA and human pathogenic dsDNA (31, 32). These limitations are consistent with prior reports (30–60% sensitivity) and underscore IIF’s role as a confirmatory tool rather than a screening method (31, 33, 34). In contrast, CLIA demonstrated higher sensitivity (41.57%) due to its chemiluminescent amplification of signals, allowing for the detection of very low concentrations of anti-dsDNA antibodies (34). CLIA’s use of recombinant or synthetic DNA as the antigen enhances its sensitivity, allowing for the detection of a broader range of antibody subtypes (35). However, due to the potential for cross-reactivity with recombinant DNA and non-specific binding, CLIA exhibits slightly lower specificity compared to IIF, which increases the risk of false-positive results (21, 36, 37).

DLCM, a more recent innovation, utilizes microfluidic chips integrated with highly sensitive detection systems to simultaneously and quantitatively measure multiple biomarkers from a single sample (18, 38). However, there is limited research on its clinical application value. Our results suggest that DLCM exhibits the highest sensitivity, likely due to its digital signal processing, which allows for the detection of small binding signals that traditional methods such as IIF may miss. This high sensitivity, combined with the digital precision of the measurement, makes DLCM a promising tool for early-stage SLE diagnosis. However, its lower specificity compared to IIF may be attributed to similar factors as those observed in CLIA, such as non-specific binding and cross-reactivity.

We also observed a high degree of consistency between DLCM and CLIA (κ = 0.78), both of which are automated, high-throughput methods. While CLIA amplifies signals through chemiluminescence, DLCM uses digital signal processing to precisely measure minute binding interactions. Our findings suggest that these two methods may be considered interchangeable in clinical practice, particularly for high-sensitivity applications. IIF’s unique detection profile (κ = 0.65–0.66 with DLCM/CLIA) reinforces its value in confirming high-affinity antibodies.

Achieving both high sensitivity and specificity with a single method is challenging, which underscores the value of combining assays to optimize diagnostic performance—using a highly sensitive method for screening and a highly specific method for confirmation (20). Our results demonstrate that combining any two methods yields better sensitivity than using a single method alone. Dual testing (e.g., IIF+DLCM) increased sensitivity to 52.23% (p < 0.001) while maintaining specificity >95%. This aligns with clinical practices in China. In fact, the combination of IIF and CLIA is typically used simultaneously for initial diagnosis, and for confirmed patients, CLIA/ELISA is employed for quantitative follow-up. In China, two methods (IIF + CLIA) are typically used simultaneously for initial diagnosis, and for confirmed patients, CLIA/ELISA is employed for quantitative follow-up. DLCM is a new automated testing method, but there is limited research on its clinical application value.

The association of anti-dsDNA antibody levels with disease activity and renal involvement underscores their prognostic value. DLCM-derived anti-dsDNA levels correlated strongly with SLEDAI scores (R = 0.42, p < 0.001), outperforming CLIA (R = 0.29, p = 0.015), highlighting its utility in tracking disease activity. Additionally, IIF and CLIA differentiated renal involvement (p < 0.05 and p < 0.001, respectively), whereas DLCM showed no significant difference (p = 0.132), possibly due to its broader antibody detection range. Dual assays improved LN detection sensitivity (e.g., IIF+DLCM: 56.64% vs. IIF alone: 48.95%, p = 0.001) but reduced specificity (53.73% vs. 69.02%), emphasizing a trade-off between early intervention and false positives. Previous studies have shown the utility of anti-dsDNA in detecting concurrent disease activity (10, 29, 39, 40) and its association with lupus nephritis (41). While several studies report a link between anti-dsDNA levels and disease activity but not renal involvement (42, 43), others suggest no correlation with either (44). Further research is needed to better understand how different testing methods may contribute to improved diagnosis and monitoring strategies.

Our study has several limitations. First, this was a single-center study, which may limit the generalizability of our findings to broader populations. Due to the heterogeneity in detection methods for anti-dsDNA antibodies across clinical laboratories, significant inter-laboratory variability in test results may arise. Future multi-center studies should validate our findings across diverse cohorts, standardize inter-laboratory protocols. Second, further research is required to explore how these assays correlate with more complete clinical manifestations of SLE. It would provide valuable insights into the role of anti-dsDNA antibody assays in disease monitoring and prognosis. Finally, our study did not incorporate cost-effectiveness analyses to compare the resource burden of these assays, which is a critical consideration for real-world implementation.

In conclusion, this study demonstrates that combining the specificity of IIF with the sensitivity of CLIA and DLCM optimizes the diagnosis and monitoring of SLE. DLCM, as a novel high-throughput automated assay, shows particular promise for initial screening and disease monitoring. Moreover, the use of combined methods enhances sensitivity for detecting renal involvement, which may facilitate earlier intervention.

## Data Availability

The raw data supporting the conclusions of this article will be made available by the authors, without undue reservation.
