# Correction: Comparative evaluation of three anti-dsDNA antibody detection methods in systemic lupus erythematosus: insights from a large monocentric cohort

**DOI:** 10.3389/fimmu.2025.1648472

**Published:** 2025-07-03

**Authors:** Ruijing Lu, Rui Yu, Rong Huang, Chiyuan Xue, Ning Song, Jiuliang Zhao, Xiaofeng Zeng, Chaojun Hu

**Affiliations:** ^1^ Department of Rheumatology and Clinical Immunology, Peking Union Medical College Hospital, Chinese Academy of Medical Sciences, Peking Union Medical College, National Clinical Research Center for Dermatologic and Immunologic Diseases (NCRC-DID), Ministry of Science & Technology, Key Laboratory of Rheumatology and Clinical Immunology, Ministry of Education, Beijing, China; ^2^ Department of Laboratory Medicine, Shenzhen Baoan Women’s and Children’s Hospital, Shenzhen, China; ^3^ Eight-year Medical Doctor Program, Chinese Academy of Medical Sciences & Peking Union Medical College, Beijing, China; ^4^ Department of Clinical Laboratory, The People’s Hospital of Yuhuan, Taizhou, Zhejiang, China

**Keywords:** anti-double-stranded DNA, systemic lupus erythematosus, indirect immunofluorescence assay, digital liquid chip method, chemiluminescence immunoassay

In the published article, an author name was incorrectly written as “Jiulang Zhao”. The correct spelling is “Jiuliang Zhao”.

The authors apologize for this error and state that this does not change the scientific conclusions of the article in any way. The original article has been updated.

