# Supplementary material for: Comparative evaluation of three anti-dsDNA antibody detection methods in systemic lupus erythematosus: insights from a large monocentric cohort
Source: Front Immunol. 2025 Apr 10;16:1529484. doi: 10.3389/fimmu.2025.1529484 (PMC12018382; doi:10.3389/fimmu.2025.1529484)
Supplement: Supplementary file 1 [file Table1.docx]

Supplementary Material

# Supplementary Table 1

**Supplementary Table 1.** ANA titers across SLE patients.

| **Patterns** | **Total, n (%)** | **Titers** | | | | |
| --- | --- | --- | --- | --- | --- | --- |
|  |  | **1:80** | **1:160** | **1:320** | **1:640** | **1:1280** |
| **Speckled** | 878 (49.52) | 110 | 166 | 292 | 199 | 111 |
| **Homogeneous** | 489 (27.58) | 46 | 88 | 148 | 126 | 81 |
| **Nucleolar** | 17 (0.96) | 1 | 8 | 7 | 1 | 0 |
| **Cytoplasmic** | 27 (1.52) | 10 | 11 | 4 | 2 | 0 |
| **Homogeneous-Speckled** | 270 (15.23) | 32 | 45 | 124 | 50 | 19 |
| **Speckled-Nucleolar** | 15 (0.85) | 5 | 8 | 2 | 0 | 0 |
| **Homogeneous-Nucleolar** | 14 (0.79) | 5 | 8 | 1 | 0 | 0 |
| **Other rare patterns*** | 3 (0.17) | 2 | 0 | 1 | 0 | 0 |
| **Negative** | 60 (3.38) | / | / | / | / | / |

** Other rare patterns include nuclear membrane, ring (rod) shape, and myosin (myofibrillar).*

# Supplementary Table 2

**Supplementary Table 2.** Statistical comparisons of sensitivity, specificity and accuracy between different methods using McNemar's test.

| **Comparison** | **Sensitivity p-value** | **Specificity p-value** | **Accuracy p-value** |
| --- | --- | --- | --- |
| IIF vs DLCM | <0.001^*^ | <0.001^*^ | <0.001^*^ |
| IIF vs CLIA | 0.016^*^ | <0.001^*^ | 0.016^*^ |
| DLCM vs CLIA | 0.032^*^ | 0.729 | 0.032^*^ |
| IIF+DLCM vs IIF | <0.001^*^ | <0.001^*^ | <0.001^*^ |
| IIF+DLCM vs DLCM | <0.001^*^ | <0.001^*^ | <0.001^*^ |
| IIF+DLCM vs CLIA | <0.001^*^ | 0.227 | <0.001^*^ |
| IIF+CLIA vs IIF | <0.001^*^ | <0.001^*^ | <0.001^*^ |
| IIF+CLIA vs DLCM | <0.001^*^ | 0.047 | <0.001^*^ |
| IIF+CLIA vs CLIA | <0.001^*^ | <0.001^*^ | <0.001^*^ |
| IIF+CLIA vs IIF+DLCM | 0.092 | 0.628 | 0.092 |
| IIF+DLCM+CLIA vs IIF | <0.001^*^ | <0.001^*^ | <0.001^*^ |
| IIF+DLCM+CLIA vs DLCM | <0.001^*^ | <0.001^*^ | <0.001^*^ |
| IIF+DLCM+CLIA vs CLIA | <0.001^*^ | <0.001^*^ | <0.001^*^ |
| IIF+DLCM+CLIA vs IIF+DLCM | <0.001^*^ | <0.001^*^ | <0.001^*^ |
| IIF+DLCM+CLIA vs IIF+CLIA | <0.001^*^ | <0.001^*^ | <0.001^*^ |

** Denotes that the comparison is statistically significant.*

*Abbreviations: IIF, indirect immunofluorescence; CLIA, chemiluminescence immunoassay; DLCM, digital liquid chip method.*

# Supplementary Table 3

**Supplementary Table 3.** Statistical comparisons of sensitivity, specificity and accuracy between different methods in SLE patients with renal involvement using McNemar's test.

| **Comparison** | **Sensitivity p-value** | **Specificity p-value** |
| --- | --- | --- |
| IIF vs DLCM | 0.433 | <0.001^*^ |
| IIF vs CLIA | 0.239 | 0.022^*^ |
| DLCM vs CLIA | 0.617 | 0.083 |
| IIF+DLCM vs IIF | 0.001^*^ | <0.001^*^ |
| IIF+DLCM vs DLCM | <0.001^*^ | 0.002^*^ |
| IIF+DLCM vs CLIA | <0.001^*^ | 0.001^*^ |
| IIF+CLIA vs IIF | 0.002^*^ | <0.001^*^ |
| IIF+CLIA vs DLCM | 0.002^*^ | 0.262 |
| IIF+CLIA vs CLIA | <0.001^*^ | <0.001^*^ |
| IIF+CLIA vs IIF+DLCM | 0.655 | 0.577 |
| IIF+DLCM+CLIA vs IIF | <0.001^*^ | <0.001^*^ |
| IIF+DLCM+CLIA vs DLCM | <0.001^*^ | <0.001^*^ |
| IIF+DLCM+CLIA vs CLIA | <0.001^*^ | <0.001^*^ |
| IIF+DLCM+CLIA vs IIF+DLCM | 0.157 | <0.001^*^ |
| IIF+DLCM+CLIA vs IIF+CLIA | 0.083 | <0.001^*^ |

** Denotes that the comparison is statistically significant.*

*Abbreviations: IIF, indirect immunofluorescence; CLIA, chemiluminescence immunoassay; DLCM, digital liquid chip method.*
